# The Urogenital System Microbiota: Is It a New Gamechanger in Urogenital Cancers?

**DOI:** 10.3390/microorganisms13020315

**Published:** 2025-02-01

**Authors:** Gülfem Ece, Ahmet Aktaş, Ayse Caner, İmran Sağlık, Tuğba Kula Atik, Özlem Ulusan Bağcı, Fulya Bayındır Bilman, Hadiye Demirbakan, Seda Güdül Havuz, Esra Kaya, Özlem Koyuncu Özyurt, Gülay Yetkin, Orçun Zorbozan

**Affiliations:** 1 Department of Medical Microbiology, İzmir City Hospital, İzmir 35540, Türkiye; gulfem.ece@gmail.com (G.E.); f_bilman@hotmail.com (F.B.B.); 2 İstanbul Provincial Health Directorate, Istanbul Public Health Laboratory No. 2, İstanbul 34524, Türkiye; ahmet.aktas@ymail.com; 3 Department of Parasitology, Faculty of Medicine, Department of Basic Oncology, Institute of Health Sciences, Ege University, Izmir 35100, Türkiye; 4 Department of Medical Microbiology, Faculty of Medicine, Uludag University, Bursa 16059, Türkiye; imransaglik@uludag.edu.tr; 5 Department of Microbiology, Faculty of Medicine, Balıkesir University, Balıkesir 10145, Türkiye; tkulaatik@gmail.com; 6 Department of Parasitology, Faculty of Medicine, Ankara University, Ankara 06230, Türkiye; drozlemulusan@gmail.com; 7 Department of Medical Microbiology, Faculty of Medicine, Sanko University, Gaziantep 27090, Türkiye; demirbakan78@yahoo.com; 8 Samsun Provincial Health Directorate, Samsun Bafra State Hospital, Department of Medical Microbiology, Samsun 55400, Türkiye; drsedahavuz@hotmail.com; 9 Department of Medical Microbiology, Kahramanmaraş Necip Fazıl City Hospital, Kahramanmaraş 46100, Türkiye; esra_ytn@hotmail.com; 10 Department of Medical Microbiology, Faculty of Medicine, Akdeniz Univertsity, Antalya 07070, Türkiye; ozlemozyurt@akdeniz.edu.tr; 11 Bakırköy Dr Sadi Konuk Education and Research Hospital, Hamidiye Faculty of Medicine, Health Science University, İstanbul 34140, Türkiye; gulayyetkin03@gmail.com; 12 Department of Medical Microbiology, Faculty of Medicine, Bakircay University, İzmir 35665, Türkiye; orcun.zorbozan@bakircay.edu.tr

**Keywords:** urogenital microbiota, dysbiosis, urogenital cancers

## Abstract

The human microbiome, which encompasses microbial communities and their genetic material, significantly influences health and disease, including cancer. The urogenital microbiota, naturally present in the urinary and genital tracts, interact with factors such as age, lifestyle, and health conditions to affect homeostasis and carcinogenesis. Studies suggest that alterations in this microbiota contribute to the development and progression of genitourinary cancers, emphasizing the concept of oncobiome, which refers to microbial genetic contributions to cancer. Similarly, gut microbiota can influence hormone levels and systemic inflammation, impacting cancers such as cervical and prostate cancer. Advanced studies indicate that microbial communities in genitourinary cancers have distinct profiles that may serve as diagnostic biomarkers or therapeutic targets. Dysbiosis of the urinary microbiota correlates with bladder and kidney cancer. Additionally, gut microbiota influence the effectiveness of cancer treatments. However, further research is necessary to clarify causality, the role of microbial metabolites, and hormonal regulation. The aim of this review is to understand that these dynamics present opportunities for innovative cancer diagnostics and therapies, highlighting the need for integration of microbiology, oncology, and genomics to explore the role of microbiota in genitourinary cancers. For this, a comprehensive search of relevant databases was conducted, applying specific inclusion and exclusion criteria to identify studies examining the association between microbiota and urogenital cancers. Research into the mechanisms by which microbiota influence urogenital cancers may pave the way for new diagnostic and therapeutic approaches, ultimately improving patient outcomes.

## 1. Introduction

The complex and diverse microbiota of the human body significantly impact human pathophysiology. Recent studies indicate that diverse microbial communities play crucial roles in maintaining host homeostasis and may even contribute to cancer development [1]. Urogenital microbiota comprise microorganisms naturally present in the urinary and genital tracts. Current evidence suggests that healthy individuals possess a substantial microbial presence in the genitourinary tract, which can be influenced by various factors such as age, gender, hormonal status, lifestyle, and both healthy and diseased states [2,3]. Genitourinary cancers, including prostate, bladder, cervical, and vaginal cancers, rank among the deadliest diseases globally [4]. Despite significant progress in prevention and treatment over recent years, the exact etiology and underlying mechanisms of these cancers remain poorly understood [5].

Furthermore, emerging data suggest that various microbial species and alterations in the genitourinary microbiota may have hematogenous potential [6,7]. A growing body of research underscores the potential role of microorganisms, with certain species of viruses, bacteria, fungi, and parasites now classified as human carcinogens. Notably, human papillomavirus has been linked to several cancers, including cervical, vaginal, and vulvar cancers, while *Chlamydia trachomatis*, a bacterium responsible for sexually transmitted infections, is also implicated [7,8]. Dysbiosis in the urinary and genital microbiota has been increasingly associated with various cancers, including bladder, kidney, cervical, ovarian, and endometrial cancers. In the case of bladder cancer, microbial imbalances are often co-etiologically associated with chronic inflammation and environmental risk factors such as smoking and *Schistosoma* infections [9]. In ovarian and endometrial cancers, changes in the vaginal microbiota, such as a reduction in *Lactobacillus* dominance, are associated with increased microbial diversity and higher malignancy risks [10]. Moreover, the concept of the “oncobiome” has emerged to describe the role of microbial genetic material in cancer initiation and progression, emphasizing the potential of microbiota as diagnostic biomarkers and therapeutic targets. Additionally, the gut microbiota introduces a systemic dimension to these interactions, influencing genitourinary cancers through hormone regulation, immune modulation, and metabolic activity. For instance, short-chain fatty acids produced by gut microbes have been associated with prostate cancer progression, and microbial metabolites are increasingly recognized for their role in modulating treatment responses in kidney cancer patients [11]. Despite these advancements, significant gaps remain in our understanding of the causal relationships between microbiota dysbiosis and cancer. The potential for leveraging the microbiome to develop novel diagnostics and therapies is immense, but realizing this potential will require interdisciplinary collaboration across microbiology, oncology, and genomics.

This review aims to explore the intricate connections between the genitourinary microbiota and cancer, highlighting recent discoveries and their implications for prevention, diagnosis, and treatment. By elucidating these complex interactions, we can pave the way for innovative approaches that may transform cancer care and improve patient outcomes.

## 2. Methods

This systematic review aimed to comprehensively address the changes in microbiota in urogenital system cancers. A comprehensive literature search was conducted using PubMed, Scopus, and Web of Science databases, with inclusion criteria encompassing peer-reviewed articles published up to September 2024 that examined microbiota profiles in urogenital cancers. Exclusion criteria included studies lacking microbiota analysis or those focused solely on non-cancerous conditions. While priority was given to research articles containing data based on the NGS method, different review articles were also used. The reference lists of the articles were also checked to avoid duplication and to identify additional studies. The search utilized keywords relevant to the topic, including types of urogenital cancers, urogenital system, microbiota, microbiome, dysbiosis, oncobiome, and urobiome. This comprehensive approach ensured a robust collection of literature relevant to the microbiota, providing a solid foundation for understanding its implications in urogenital cancers.

## 3. Pathogenesis of Cancer

The urogenital microbiota plays a crucial role in maintaining the health and homeostasis of the urogenital system. In women’s urogenital systems, the vaginal microbiota consists primarily of species that help maintain a low pH and provide protection against pathogens, and it is well characterized. However, the microbiota in the urinary tract and prostate in men are less well characterized [12,13,14].

The dysbiotic microbiome, which causes an inflammatory response, disrupts the integrity of epithelial cells [15]. Imbalance or dysbiosis can lead to epithelial barrier dysfunction [15], triggering inflammatory responses [15,16,17], inducing genomic effects [18], and disrupting metabolic and hormonal processes [19,20]. Furthermore, the concept of the “oncobiome” has emerged to highlight the significance of the relationship between the genetic material of microbial communities and oncogenesis, defining the role of the microbiome in cancer initiation and progression [21]. These alterations may contribute to a tumor-permissive microenvironment and affect the cancer process [20,22,23].

Carcinogenic viruses utilize various mechanisms to contribute to the development of cancer cells. For instance, HPV expresses well-characterized oncoproteins (E6 and E7) that promote cell proliferation, prevent cell death, and/or induce genetic instability [24]. The association of HPV E6 and E7 oncoproteins with cancer is well-documented in scientific literature. The protein encoded by the E6 oncogene targets the p53 protein, causing cellular changes that may lead to the development of malignant tumors. The E7 protein affects the retinoblastoma protein, leading to the release of E2F and the initiation of DNA synthesis [18]. Apolipoprotein B mRNA editing enzyme catalytic polypeptide-like 3 (APOBEC3) serves as the source of DNA mutations induced as a cell-intrinsic response to infection. Cells induce the expression of APOBEC3 to mutate and inactivate invading viral genomes. However, these enzymes also cause collateral damage in the form of point mutations in the cellular genome, which can lead to cancer formation. Consistent with this, a significant proportion of cellular mutations in HPV-associated cancers have an APOBEC3 mutational signature [25].

Cervicovaginal dysbiosis and bacterial vaginosis can increase susceptibility to infection with oncogenic HPV genotypes, leading to genital inflammation and cervical neoplasia. Maarsingh et al. [26] integrated bacterial vaginosis-associated microorganisms, such as *Fusobacterium*, *Lancefieldella*, *Peptoniphilus*, and *Porphyromonas*, into a three-dimensional (3D) cervical epithelial cell culture model to investigate their association with gynecologic neoplasia in vitro. They showed that *Lancefieldella parvulum* and *Peptoniphilus lacrimalis* elicited robust pro-inflammatory responses in 3-D cervical cells, while *Fusobacterium nucleatum* and *F. gonidiaformans* modulated metabolic hallmarks of cancer leading to the accumulation of 2-hydroxyglutarate, pro-inflammatory lipids, and signs of oxidative stress and genotoxic hydrogen sulfide. This research provides insights into how gynecological cancer-associated bacteria may create a tumor-promoting microenvironment in the human cervix [26].

Chronic inflammation is a well-established risk factor for carcinogenesis [27]. Specifically, an overabundance of pathogenic bacteria could trigger an inflammatory reaction, producing cytokines and other mediators that may contribute to tumorigenesis. Walther-Antonio et al. [28] reported that co-colonization of *Fannyhessea vaginae* (formerly *Atopobium vaginae*) with *Porphyromonas* sp. was associated with endometrial cancer. Based on this research, Caselli et al. [29] showed that in an in vitro model, *F. vaginae* and *Porphyromonas somera* increased the release of inflammatory cytokines and chemokines (IL-1α, IL-1β, IL-17α, and TNF-α) in endometrial cells, indicating their role in maintaining inflammation and initiating neoplastic processes. This was supported by another study demonstrating a link between dysbiosis, high inflammatory cytokine expression, and endometrial cancer [17]. They found that the abundance of *Micrococcus* was associated with IL-6 and IL-17 mRNA levels in endometrial cancer. The inflammatory response associated with infection by *C. trachomatis* can lead to increased reactive oxidative metabolites; increased expression of cytokines, chemokines, and growth and angiogenic factors; decreased cell-mediated immunity; and the generation of free radicals and their ability to cause DNA damage. In addition, the ability of the bacterium to disrupt DNA repair and host cell cycle control may lead to genetic instability and a favorable environment for malignant transformation. Moreover, coinfection of HPV and *C. trachomatis* has a higher risk of cervical cancer [30].

In addition to inflammatory responses, bacterial toxins and metabolites such as nitrosamines are potential mechanisms by which the microbiota can influence carcinogenesis by causing genotoxic effects. Pathogen-associated molecular patterns in chronic infections can directly lead to bladder cancer by activating inflammatory pathways that result in cellular mutations and subsequent malignancy [31]. Persistent bacterial infection of the prostate causes chronic inflammation, which promotes DNA damage through the production of leukocyte-derived active oxygen and nitrogen species. These may either directly cause cancer or worsen its progression [27]. Some microbial metabolites, like nitrosamines, have been associated with the development of bladder cancer. Infections such as *Schistosoma haematobium* infection have an oncogenic effect on bladder cancer, stimulating N-nitrosamine synthesis and sustaining chronic inflammatory induction [32].

Hormone metabolism, especially estrogen and testosterone, plays a significant role in genitourinary cancers. Estrogen-like metabolites of *S. haematobium* soluble egg antigen have been found to trigger important cancer characteristics, such as enhanced cell proliferation and decreased apoptosis [19,33]. Microorganisms can not only trigger cancer but also impact the cancer process by affecting the mechanisms responsible for the immune system. *Lactobacillus* dominance decreased with the severity of cervical neoplasm, which correlated with elevated vaginal pH [34].

As a distant microbial community, changes in the intestinal microbiota can impact hormone levels and increase the risk of hormone-related cancers such as cervical, ovarian, and prostate cancers. Studies have shown that the intestinal microbiota may affect cancer development by producing a variety of metabolites and hormones that have systemic effects on the genitourinary system [35,36,37]. The gut microbiome can produce products capable of metabolizing estrogens and regulating estrogen levels [38,39]. It can also lead to systemic inflammation, a crucial factor in cancer development. Similarly, there are suggestions that changes in the genital microbiota may affect hormone levels, influencing the risk of hormone-related cancers [34,40,41]. Although the effect of the intestinal microbiome on hormones has been observed in a few studies to date, further investigation is required to determine how the genitourinary microbiome affects genitourinary cancers through the hormones responsible for different cancers in men and women.

## 4. Urinary System Microbiota

The urinary microbiota (urobiome) refers to the community of microorganisms found in the urinary tract (kidneys, ureters, bladder, and urethra). Similar to the gut microbiota, urobiome studies have shown that different sampling techniques for urine have varying diversity of microorganisms [42]. These microorganisms can influence urinary tract health, and their dysbiosis has been implicated in various urogenital conditions, including urinary tract infections (UTIs), interstitial cystitis, urgency urinary incontinence, urinary tract cancers, and even sexual health [43,44].

A recent study has revealed differences between the bacteria detected in urine samples and those found in bladder mucosal tissue samples [45]. The 16S rRNA sequencing-based urine microbiome shows similarities at the phylum level for both men and women, with the majority of bacteria belonging to the Bacillota phylum (65% in men, 73% in women). The remaining phyla make up a total of 97%, and these are ranked as Actinomycetota (15% in men, 19% in women), Bacteroidota (10% in men, 3% in women), and Pseudomonadota (8% in men, 3% in women) [46,47].

The midstream urine microbiota of healthy women consist of highly diverse microbiota and are dominated by *Lactobacillus*, *Gardnerella*, *Sneathia*, *Staphylococcus*, and *Enterobacteriaceae* [47]. Studies on the male urinary microbiota are quite limited, and genera such as *Lactobacillus*, *Sneathia*, *Veillonella*, *Corynebacterium*, *Prevotella*, *Streptococcus*, and *Ureaplasma* are commonly found in urethral swabs of healthy men [48,49]. However, significant differences between the male and female urobiomes have also been reported [50]. Notably, the female urinary microbiota is dominated by *Lactobacillus*, while the male microbiota is dominated by *Corynebacterium* and *Streptococcus* [46].

Although most studies have focused on bacteria, the presence of fungi, viruses, and archaeal species has also been identified in the urobiome. The fungal community in the urinary tract, particularly in healthy individuals, has not been sufficiently characterized [51]. The urinary fungal community is primarily composed of individuals from the classes *Dothideomycetes*, *Saccharomycetes* (the class to which *Candida* belongs), *Eurotiomycetes*, *Exobasidiomycetes*, and *Microbotryomycetes* [52]. On the other hand, the viral community in the urinary tract is primarily composed of bacteriophages, although some eukaryotic viruses have also been identified [51].

Dysbiosis, the imbalance of gut microbiota, can severely disrupt the intestinal barrier, increasing intestinal permeability and leading to a condition commonly referred to as leaky gut. This breach in the gut lining allows pathogenic microorganisms, toxins, and harmful substances to enter the bloodstream, triggering an inflammatory immune response [53]. Chronic low-grade inflammation, often a consequence of dysbiosis, has been established as a key contributor to cancer progression, creating a tumor-promoting microenvironment [54]. Inflammatory cytokines and immune cells activated by dysbiosis can further support tumor development by promoting cell proliferation, survival, and angiogenesis [55]. Moreover, this inflammation can impair the immune system’s ability to recognize and eliminate cancerous cells [56]. Additionally, dysbiosis influences hormone metabolism, particularly sex hormones like estrogen, which can heighten the risk of hormone-dependent cancers, such as ovarian cancer, by disrupting estrogen regulation [57]. Gut microbiota play a crucial role in modulating estrogen levels via the enterohepatic circulation, and dysbiosis can lead to elevated estrogen levels, further increasing the risk of estrogen-driven cancers [58]. Furthermore, imbalances in the gut microbiota can affect the gut–liver axis, disrupting the regulation of thyroid hormones and cortisol, which may alter cellular function, metabolism, and stress responses, thereby contributing to cancer development [59]. Some gut microbes also produce genotoxic metabolites that can damage DNA, leading to mutations, chromosomal instability, and eventually cancer [60]. Such genotoxic metabolites are often overproduced in dysbiosis, heightening the risk of cancer through direct DNA damage and impairing the body’s ability to detoxify harmful substances [61]. In sum, dysbiosis can contribute to cancer development through a multitude of mechanisms, including immune dysregulation, hormonal imbalances, and direct genetic damage.

**Figure 1:**
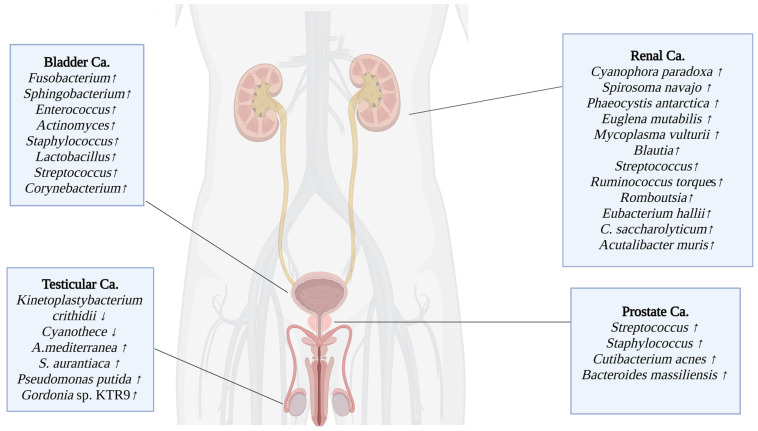
Changes in microbiota composition in cancers of the urinary system and male genital system. Arrows in the figure show increases or decreases in bacterial abundance. ↑: Relative abundance increase; ↓: Relative abundance decrease.

Dysbiosis in the urobiome is associated with various urological disorders, including benign conditions and overactive bladder. Additionally, there is emerging evidence linking urobiome dysbiosis to prostate cancer, particularly in cases with a history of recurrent antibiotic use. A case-control study has shown that regular probiotic intake in healthy individuals may reduce the risk of bladder cancer [44], thus suggesting a potential relationship between urobiome content and bladder cancer [43]. Recent studies on the urobiome have identified potential correlations between dysbiosis in the urobiome and the development and persistence of urological cancers, similar to relationships observed in the gut microbiome [62] (Figure 1).

Research on the urobiome holds significant opportunities for enhancing our understanding of urinary system diseases and can pave the way for new diagnostic and therapeutic approaches through microbiota analysis.

### 4.1. Renal Cancer

Renal cell carcinoma (RCC) is ranked as the 14th most common neoplasm worldwide [63], with an incidence rate of 4.5 cases per 100,000 people. Renal cell carcinoma is a type of cancer originating from the renal parenchyma, highly resistant to chemotherapy and causing distant metastases. In general, risk factors associated with this disease include smoking, obesity, and hypertension [62]. Emerging evidence suggests that the microbiota of the gastrointestinal and genitourinary systems may serve as a contributing risk factor in the development of RCC due to differences in microbial diversity and metabolism [62,64].

Although urine is known to be sterile, recent evidence supports the existence of a urinary microbiome [62,64]. While *Lactobacillus* spp. and *Gardnerella* spp. are particularly dominant in the female urinary microbiota, *Corynebacterium* spp., *Staphylococcus* spp., and *Streptococcus* spp. constitute the majority of the male urinary microbiota. This microbiota difference is correlated with the higher incidence of genitourinary carcinomas observed in men [23,64,65]. Notably, the diversity of urinary microbiota of RCC patients has been reported to differ from that of healthy volunteers [62,66].

A study aimed at profiling the intratumoral microbiota in renal tumors revealed that species diversity was diminished in RCC tissues, with an increase in 25 taxa and a decrease in 47 taxa when compared to adjacent normal tissues. Specifically, *Chloroplast* and the *Streptophyta* taxa exhibited strong indicator accuracy for distinguishing RCC tissues from normal tissues [67].

Recent studies on the renal microbiome have shown promising insights. Heidler et al. [68] evaluated five patients with kidney cancer following radical nephrectomy, comparing neoplastic renal tissue with adjacent normal tissue in terms of microorganisms. The study utilized 16S rRNA sequencing on ten biopsy materials obtained from patients who had undergone laparoscopic nephrectomy without urinary tract infection history in the prior 6 months. The results showed a significant difference between benign and malignant renal tissues (*p* < 0.0001). The following microorganisms were found in healthy tissue only: Terrabacteria, Stenosarchaea, *Microbacterium* spp., *Pelomonas* spp., *Staphylococcus* spp., *Leuconostoc garlicum*, *C. vitaeruminis*, *Anaerococcus nagyae*, *Ethanoligenens harbinense*, *Neisseria bacilliformis*, *Thermicanus aegyptius*, and *L. mesenteroides*. Microorganisms that appeared in cancer tissue only were *Cyanophora paradoxa*, *Spirosoma navajo*, *Phaeocystis antarctica*, *Euglena mutabilis*, and *Mycoplasma vulturii*. The abundance of microorganisms in renal cancerous tissue was found to be four times higher than that in the adjacent normal tissue. In a similar study, Kovaleva et al. [69] identified Actinomycetota, Pseudomonadota, Bacillota, Cyanobacteriota, and Bacteroidota as the dominant microorganisms in both normal and malignant kidney tissues, noting specific bacterial populations associated with different histotypes of renal tumors.

Research on the impact of microbiomes on kidney cancers has mostly focused on the gut microbiota [70,71,72]. Pathogenic microorganisms are known to contribute to carcinogenesis through chronic inflammatory response, either directly or indirectly with their metabolites [62,64]. A study conducted in China found a significant difference between the intestinal microbiota composition of RCC patients and healthy volunteers, highlighting the frequent presence of *Blautia* spp., *Streptococcus* spp., *Ruminococcus torques* group, *Romboutsia* spp., and *Eubacterium hallii* group bacteria in RCC patients. These five species have been proposed as potential biomarkers for RCC, with *S. lutetiensis* identified as particularly important in the progression and metastasis of RCC [62,73].

Ecological imbalance of the oral microbiome, the initial part of the digestive system, is the primary factor leading to the development of periodontitis [62]. While some studies suggest a correlation between periodontitis and an increased risk of kidney cancer, others report no such association [62,74,75]. These contradictory results may vary depending on regional differences as well as the methodologies utilized in microbiota studies [62,64].

The gut microbiota also influences the efficacy of RCC treatments, including chemotherapy and immunotherapy [62,64,76]. Specifically, gut microbiota may serve as a predictor for treatment outcomes in RCC patients. Patients who benefit from immunotherapy treatment have gut microbiota with higher microbial diversity [62,77]. In animal model studies using fecal transplants from responsive patients, the microbiota has been shown to modulate the response to anti-PD-1 therapy [64]. Notable microbial taxa associated with favorable treatment responses include Bacillota, *Akkermansia muciniphila*, and *Bifidobacterium adolescentis*, among others [62,77,78]. The species that may mediate the relationship between immunotherapy and treatment response is *A. muciniphila* [76], an anaerobic bacterium that lives in the intestinal mucosa, which is particularly notable for its role in increasing microbial diversity and inhibiting pathogenic bacteria [62]. In vitro studies have demonstrated that *A. muciniphila* secretes different substances that inhibit tumor growth and increase treatment response [62,79]. Furthermore, the use of antibiotics is known to disrupt the gut microbiota and cause suboptimal treatment responses in RCC patients treated with immunotherapy [62].

The composition of gastrointestinal microbiota may also change during RCC treatments, with pathogenic shifts associated with treatment-related toxicities being observed [64]. Diarrhea is a common and potentially dose-limiting toxicity of anti-vascular endothelial growth factor (anti-VEGF) therapy used in RCC treatment. High levels of *Bacteroides* spp. and low levels of *Prevotella* spp. were detected in stool microbiota profiles from RCC patients experiencing diarrhea during this treatment [64,80]. It is posited that microbiota may be useful as an adjuvant therapy to increase the therapeutic effects of treatments used for RCC [62].

In a study on dysbiosis of gut microbiota, the *Ruminococcus torques* group and the *Erysipelatoclostridium* spp. were associated with an increased risk of renal carcinoma [71]. Additionally, studies have shown associations between RCC and changes in gut microbiota and tryptophan metabolites. Dai et al. [81] reported that the disturbance of tryptophan metabolites in the gut microbiota is associated with renal cancer metastasis, whereby activation of aryl hydrocarbon receptor (AhR) further promoted migration and invasion of RCC cells and inhibited apoptosis of RCC cells. This study reported that the composition of the gut microbiota was significantly altered in RCC patients, and *Bacteroides* spp. and *Akkermansia* spp. were significantly increased, while *Blautia* spp., *Bifidobacterium* spp., and *Megamonia* spp. were significantly decreased. These microbiota changes have also been observed to affect the success of immune-mediated therapies in RCC, with tryptophan catabolism implicated in tumor progression. In a prospective study involving 69 patients, Derosa et al. [82] showed that in metastatic RCC patients treated with nivolumab, a higher response rate was associated with increased levels of *B. salyersiae*, *A. muciniphila*, and *E. siraeum*, alongside decreased abundance of *C. clostridioforme* and *C. hathewayi* in the fecal microbiota.

### 4.2. Bladder Cancer

Bladder cancer (BC) is ranked 10th globally among all cancer types and is the 13th leading cause of cancer-related deaths, with 573,278 new cases and 212,536 deaths per year. It represents the second most common cancer of the urinary tract among men, following prostate cancer. The incidence of BC is notably higher in men than women (9.6 per 100,000 men vs. 2.4 per 100,000 women). Furthermore, individuals aged over 65 are particularly susceptible to this disease [83,84].

The most well-known risk factor for BC is smoking, followed by exposure to occupational chemicals, host genetics, schistosomiasis, contaminated drinking water, and other microorganisms. The infection caused by Schistosoma has long been recognized as a contributing factor to BC, particularly in endemic regions such as Egypt. Recent evidence suggests that microbiota also play a role in the relationship between Schistosoma and BC. In a study by Adebayo et al. [9], involving individuals with BC, Schistosoma-only infections, and healthy controls, *Fusobacterium*, *Sphingobacterium*, and *Enterococcus* were the predominant genera in patients with *Schistosoma*-induced BC. The study identified *Sphingobacterium* as a potential biomarker of *Schistosoma* infection, while taxa like *Trabulsiella* and *Weissella* were associated with non-infected individuals.

Based on the literature, numerous studies have attempted to elucidate connections between intestinal, urinary, and tissue microbiota and the incidence or progression of BC. A study conducted by Parra-Grande et al. [85] found that Actinomycetota was associated with a low incidence of bladder tumors, while *Barnesiella*, *Enterococcus*, *Prevotella*, *Alistipes*, *Parabacteroides*, and *Lachnospiracea*_incertae_sedis (Cluster 1) or *Staphylococcus* (Cluster 2) species were detected at higher abundance in patients with tumors. Additionally, in the study by Mansour et al. [45], tissue and urine microbiota were concurrently assessed in patients diagnosed with BC. This study found that five suspect genera—*Akkermansia*, *Bacteroides*, *Clostridium* sensu stricto, *Klebsiella*, and *Enterobacter*—were significantly more prevalent in tissue samples than in urine samples. Bacillota emerged as the most predominant phylum in both sample types. However, the prevalent genera varied across the two groups: *Klebsiella*, *Akkermansia*, *Clostridium* sensu stricto, and *Bacteroides* were more prevalent in tissue samples, while *Staphylococcus*, *Lactobacillus*, *Streptococcus*, and *Corynebacterium* exhibited higher abundance in urine samples.

The identification of microorganisms within the microbiota that are associated with cancer etiology or prognosis could potentially serve as biomarkers for early diagnosis or therapeutic targets. The utility of non-invasive samples, such as urine and stool, for the identification of microbial biomarkers further increases their diagnostic potential. In the study by Bi et al. [86], the urine samples from patients with BC showed a higher abundance of *Actinomyces* spp., whereas the samples from healthy individuals showed an enrichment of *Lactobacillus*, *Streptococcus*, *Bifidobacterium*, and *Veillonella* genera. Similarly, the study conducted by Chorbińska et al. [87] evaluated both urine and stool samples of BC patients and healthy individuals. The phylum Thermodesulfobacteriota (formerly Desulfobacterota) exhibited variations in abundance correlated with tumor grade (G) in the stool samples of BC patients, being least abundant in G2 and most abundant in G1.

The current gold standard for treating high-grade intermediate-risk and high-risk non-muscle invasive BC is the intravesical injection of *Mycobacterium bovis* Bacillus Calmette-Guerin (BCG) [88]. Numerous studies have explored both the impact of BCG treatment on microbiota and the significance of microbiota in assessing the success of BCG therapy in recipients. Patients with a history of BCG therapy had a higher prevalence of the *Lactobacillus* spp. [87]. In addition, another study showed a considerable increase in the abundance of *Brochothrix*, *Serratia*, *E. coli*, *Negativicoccus*, and *Pseudomonas* in patients who responded well to BCG therapy [89]. Furthermore, pre-treatment BCG abundance of *Streptococcus*, *Lactobacillus*, and *Cutibacterium* in bladder microbiota was linked to treatment efficacy [90].

## 5. Genital System Microbiota

The genital system microbiota refers to the diverse microbial populations residing within the genital tracts, closely connected to nearby mucosal sites. Socio-economic, behavioral, environmental, hormonal, and genetic factors can all influence the diversity of the microbiota in the male and female genital tracts. It is expected that they differ from each other due to anatomical structure differences, in addition to hormonal effects. In this context, female and male genital tract microbiota will be discussed under separate headings.

Although most studies on the female reproductive tract microbiome focus on the vagina due to ease of sampling, microbiota have also been demonstrated in the uterus, fallopian tubes, and ovaries [91]. In the majority of women of reproductive age, the microbiota of the vagina and cervix are dominated by *Lactobacillus* species (spp.), with microbiome abundance decreasing from outside to inside [92]. *Lactobacillus* spp. benefit the host through symbiotic relationships.

The amount of glycogen in the vaginal epithelial cells is increased by estradiol. *Lactobacillus* spp. metabolize this glycogen by anaerobic fermentation, forming lactic acid, which both lowers the vaginal pH in a range of 3.8 to 4.5 and ensures that it is the dominant species in the vaginal microbiota [93]. The vaginal microbiota is categorized into five community state types (CSTs) according to species composition. While CST I, II, III, and V are dominated by *Lactobacillus* spp. (*L. crispatus*, *L. gasseri*, *L. iners*, and *L. jensenii*, respectively), CST IV has higher species diversity and non-*Lactobacillus* dominance [13].

Due to possible contamination during sampling, uterine and endometrial microbiota composition is controversial [14]. The mentioned studies have shown that the most abundant genera in hysterectomized uteri are *Lactobacillus* and *Prevotella* [10].

The upper side of the female genital tract may have a distinct low-biomass microbiome. Studies on tubal microbiota composition explored using salpingectomy materials have shown the presence of *Acinetobacter*, *Comamonas*, *Pseudomonas*, *Delftia*, and *Burkholderia* spp. in phylum Pseudomonadota (formerly Proteobacteria); *Dysgomonas* and *Prevotella* spp. in phylum Bacteroidota (formerly, Bacteroidetes); *Staphylococcus*, *Enterococcus*, and *Lactobacillus* spp. in phylum Bacillota (formerly Firmicutes); and *Propionibacterium* spp. in phylum Actinomycetota (formerly Actinobacteria) as the most abundant genera [14].

**Figure 2:**
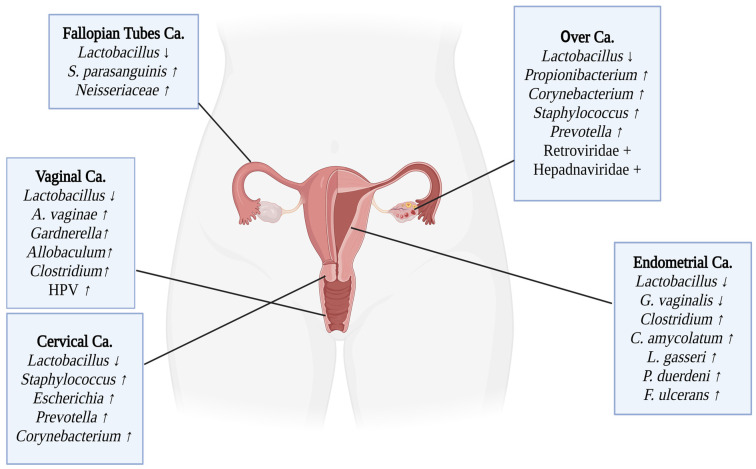
Changes in microbiota composition in gynecological cancers. Arrows in the figure show increases or decreases in bacterial abundance. ↑: Relative abundance increase; ↓: Relative abundance decrease.

Seminal fluid is used in studies investigating male genital microbiota. Studies, both in healthy donors and azoospermic patients, have shown that Acinetobacter and *Pelomonas* spp. in phylum Pseudomonadota; *Porphyromonas* and *Prevotella* spp. in phylum Pseudomonadota; *Lactobacillus*, *Enterococcus*, *Veillonella*, and *Streptococcus* spp. in phylum Bacillota; *Sneathia* spp. in phylum Fusobacteriota (formerly Fusobacteria); and *Corynebacterium* spp. in phylum Actinomycetota are the most abundant genera [14] (Figure 1 and Figure 2).

### 5.1. Ovarian Cancer

Ovarian cancer (OC) is ranked eighth among cancers and cancer-related deaths in women worldwide. According to Globocan data, approximately 320,000 new cases and 205,000 deaths from OC are reported annually [94]. OC can be caused by many factors, such as mutations in the *BRCA1* and *BRCA2* genes and hormonal factors. In addition, thanks to new generation sequencing methods, it has been shown that the microbiome also contributes to the development of OC. Furthermore, changes in the gut microbiome can influence estrogen levels by affecting enterohepatic circulation, further increasing the risk of cancer [95].

OC typically progresses asymptomatically in its early stages, leading to diagnosis at stages 2 or 3, which makes the disease more difficult to treat. Whether detecting microbiome changes could aid in early diagnosis remains a question [96]. For this reason, many studies have been conducted to clarify the relationship between the microbiome and OC. Banerjee et al. [97] detected viruses from the Retroviridae (Hepadnaviridaea, Paramyxoviridaea, Rhabdoviridaea and Togaviridaea). In terms of bacteria, Pseudomonadota was dominant in patients (52%). In addition, unlike in the control group, four different phyla (Chlamydiae, Fusobacteriota, Spirochaetota, and Mycoplasmatota) were detected. At the same time, 52 different genera of bacteria were detected in the patient group, showing that the bacterial diversity was much higher than in the control group. The most frequently detected genus in the OC group was *Pediococcus*. The most frequently detected genus among fungi was *Cladosporium*. This comprehensive study shows that the tissue with OC has its own special microbiome and that increased microbial diversity can cause OC. Miao et al. [98] found significant differences in the microbiome between the malignant tumor group and the benign tumor group, identifying 18 microbial features specific to OC. Bacteria such as *Prevotella*, *Odoribacter*, *Bacteroides*, *Clostridium*, *Bradyrhizobium*, *Sutterella*, and *Oscillospira* were detected more frequently in the OC. Jacobson et al. [99] found that in the microbiome analysis of vaginal and fecal samples of a patient with OC (n = 45) who responded differently to different treatments, *Lactobacillus*, *Prevotella*, *Echerichia*, *Gardnerella*, and *Streptococcus* dominance was found in vaginal samples, and the rate of *Prevotella* increased in fecal samples. They associated the change in the microbiome with a significant difference in treatment response. Morikawa et al. [100] showed a decrease in the density of *Lactobacillus* in patients with OC; on the contrary, *Propionibacterium*, *Prevotella*, *Corynebacterium*, *Staphylococcus*, and *Streptococcus* became dominant. Asangba et al. [101] showed a decrease in both the number and species diversity of *Lactobacillus* and an increase in *Streptococcus*, *Aerococcus*, *Veillonella*, and *Megasphaera* spp. in the vaginal and cervical microbiomes of patients with OC. The microbiomes of the uterus, ovaries, and fallopian tubes were similar in both groups, but an increased presence of *Bacteroides* was detected in those with OC. Overall, bacterial diversity was higher in the OC group at all sampled sites. Similar to the study by Morikawa et al. [100], the researchers suggested that the risk of OC in women could be assessed by evaluating the cervicovaginal microbiome in this study [100,101].

According to studies, there is a clear relationship between the decrease in the number of *Lactobacillus* in the microbiota in the female reproductive system, the increase in bacterial diversity, and ovarian cancer. This relationship can be supported by additional factors and made suitable for use in the screening of early-stage OC. There is also an important relationship between the response to treatment and the microbiome. However, many more studies are needed to determine whether these changes in the microbiome are the cause or consequence of cancer. Additionally, whether microbiome regulation will contribute to preventing OC or responding to treatment is another issue that needs to be investigated.

### 5.2. Fallopian Tube Cancer

Primary fallopian tube cancer is extremely rare, accounting for less than 1% of gynecologic malignancies. Identified risk factors include hereditary breast and ovarian cancer syndromes, *BRCA1* or *BRCA2* gene mutations, nulliparity, and advanced age typically seen in postmenopausal women [102]. Fallopian tube cancers may be misdiagnosed as ovarian carcinoma because the fallopian tube epithelium is the site of origin for a significant portion of high-grade serous carcinomas [103].

Although the upper genital tract of women has generally been considered sterile, recent studies have revealed the presence of bacteria in this region even in the absence of clinical signs of infection [10,104]. The translocation of microorganisms from the lower genital tract to the fallopian tubes may create a pro-inflammatory environment that could contribute to the carcinogenesis of the fallopian tube or ovarian epithelium. The effect of microorganisms on carcinogenesis depends on several factors, including the type of microorganism, its concentration, host-related factors, and duration of exposure. A significant change in the fallopian tube microbiota has been observed in cancer patients. Notably, changes in the fallopian tube microbiota in OC patients suggest a potential role of the microbiome in the pathogenesis of malignancies at this site [8,18,104,105]. Yu et al. [105] compared the prevalence of 84 bacterial taxa between OC patients and those without cancer. In OC patients, they found that 90% of the 20 most common bacterial species came from outside the female reproductive system, consisting primarily of gastrointestinal and oral bacteria. In contrast, bacterial species typically found in the vagina, such as *Corynebacterium amycolatum* and *L. iners*, were more common in the fallopian tubes of non-cancer patients. Additionally, in the fallopian tube, a specific bacterial species is more prevalent in the serous carcinoma subtype than in other OC subtypes. It has been suggested that infection with certain genital tract bacteria may lead to chronic tubal inflammation and cell DNA damage, potentially contributing to neoplastic transformation [104,105]. Due to limited knowledge on this topic, further studies are necessary to investigate the relationship between primary fallopian tube cancer and microbiota.

### 5.3. Endometrial Cancer

Endometrial cancer (EC) is the sixth most common cancer in women worldwide [106]. It usually occurs in postmenopausal women, and risk factors include obesity, hormonal imbalances, diabetes, and a family history of certain types of cancer [106,107]. Recent studies have suggested that changes in the vaginal and endometrial microbiota may influence the progression of EC by affecting local immune responses, inflammation, and estrogen metabolism. The inflammatory process induced by polymicrobial infection also leads to dysbiosis. It is accepted that dysbiosis is a factor that stimulates carcinogenesis [108].

The vaginal ecosystem is a complex microbial environment that allows both beneficial bacteria and opportunistic pathogens to survive and proliferate. Vaginal microbiome varies throughout a woman’s life due to menstrual status, nutrition, hygiene, antibiotic use, socio-economic status, sexual activity, number of sexual partners, alcoholism and smoking, and ethnicity [109]. It is believed that lactobacilli colonizing the vagina and cervix reduce the possibility of pathogen invasion by lowering the vaginal pH through fermentation end products, creating bacteriostatic or bactericidal substances [34,106]. Dysbiosis occurs with a decrease in lactobacilli and an increase in anaerobes [34].

When the vaginal/cervical microbiota composition of patients with EC and precancerous lesions was examined, *L. iners* was significantly more common in patients with benign disorders, while *Dialister pneumosintes* and *Mobiluncus curtisii* were found in cancer patients. Overall, *Lactobacillus* spp. and *G. vaginalis* were reported to be significantly less common in the cancer group. Recent studies have indicated a strong association between vaginal microbiome composition and survival in EC patients. In the investigations of the relationship between the microbiome and disease severity in patients with EC, Hakimjavadi et al. [110] identified *Fusobacterium ulcerans* as the only species significantly enriched in patients with high-grade EC. Besides, a significant microbiome abundance of *Clostridium* spp., *C. amycolatum*, *L. gasseri*, and *Peptoniphilus duerdeni* was found in patients with low-grade EC compared to those with benign disease.

The vaginal microbiome of healthy women is predominantly characterized by species belonging to the genus *Lactobacillus*. Specifically, the vaginal and cervical microbiome of healthy women mainly consists of *L. crispatus*, *L. gasseri*, *L. iners*, *L. jensenii*, *L. vaginalis*, and *G. vaginalis*, whereas subtypes such as *L. acidophilus* are unable to colonize the vagina effectively and establish dominance [20,34]. In asymptomatic North American women, *L. crispatus*, *L. iners*, *L. gasseri*, and *L. jensenii* were the dominant lactic acid bacteria identified, while anaerobic bacterial communities such as *Prevotella* spp., *Dialister* spp., *Atopobium* spp., *Gardnerella* spp., *Megasphaera* spp., *Peptoniphilus* spp., *Sneathia* spp., *Eggerthella* spp., *Aerococcus* spp., *Finegoldia* spp., and *Mobiluncus* spp. were observed at higher rates [111].

A study conducted by Walther-António et al. [28] reported that certain specific bacterial species from the Bacillota, Actinomycetota, Bacteroidota, and Pseudomonadota were very commonly identified in patients with EC. Notably, the presence of *Atopobium vaginae* and *Porphyromonas* spp. in samples obtained from the lower genital tract is strongly associated with the presence of EC.

Furthermore, it is known that increased diversity in the microbiota may serve as a potential risk factor for EC. Sobstyl et al. [106] have reported that *Anaerococcus tetradius*, *A. lactolyticus*, *Peptoniphilus coxii*, and *Campylobacter ureolyticus* were detected in certain samples in association with menopausal status. The presence of *Porphyromonas somerae*, as confirmed by polymerase chain reaction (PCR), has been determined as the most significant microbial marker of EC.

The colonization of Bacillota and Bacteroidota in the lower genital tract increases endogenous estrogen levels by enhancing estrogen deconjugation via β-glucuronidase and β-glucuronide enzymes. Increased estrogen levels subsequently stimulate glycogen secretion from vaginal epithelial cells. Lactobacilli metabolize this glycogen, resulting in the production of lactic acid, which maintains the vaginal pH below 4.5, thus providing a protective effect against dysbiosis [34]. Besides, elevated estrogen levels stimulate ERα receptors in the endometrium, promoting carcinogenesis through endometrial cell growth, proliferation, inhibition of apoptosis, and angiogenesis [112]. Additionally, carcinogenic mechanisms have been suggested, including destruction of the natural epithelial barrier by bacterial toxins, impairment of the immune response, damage to host DNA histones, integration of specific oncogenes into the host genome, and mutations of tumor suppressor genes (e.g., p. 53) [34].

Epidemiological evidence supports a correlation between inflammation and microbiome dysbiosis. Notable findings that suggest a relationship between the carcinogenesis of EC and chronic inflammation include increased levels of C-reactive protein, NF-κB, activation of iNOS, and increased levels of IL-6, IL-8, and IL-1Ra [113]. Furthermore, vaginal epithelial cells have been shown to secrete IL-6 and IL-8 in response to *A. vaginae* and *G. vaginalis*, but not in response to *L. crispatus*. Significantly increased mRNA expression of pro-inflammatory cytokines (e.g., IL-6, IL-8, and IL-17) has been found in women with EC compared to those with benign uterine lesions [17]. The uterine microbiome has the potential to trigger the production of pro-inflammatory cytokines in endometrial cells, such as IL-1α, IL-1β, IL-17α, and TNF-α, all of which are related to the carcinogenesis of various cancers. Endometrial cancer is recognized as estrogen-dependent, and dysbiosis in both the uterus and the intestinal microbiota is indirectly associated with carcinogenesis, as it contributes to an increase in serum estrogen levels by reactivating estrogen metabolites [106].

The maintenance of a balanced vaginal microbiome appears to be an important goal for clinicians treating women with any risk factor for gynecologic malignancy. It is important to note that the use of antibiotics alone may provide insufficient treatment for women undergoing oncologic treatment who exhibit vaginal microbiome disorders. Promising studies are being conducted with the administration of vaginal probiotic strains of lactobacilli, such as *L. crispatus*, in combination with antibiotics in patients with dysbiosis. Besides, supportive immunotherapy, when combined with primary treatment for low-grade endometrial tumors, may enhance therapeutic success [20,34,106].

### 5.4. Cervical Cancer

Cervical cancer (CC) contributes to 6%–29% of all cancers in women [114]. Cervical cancer is initiated by HPV infection, which leads to uncontrolled cellular proliferation and eventually results in cancer. Recent studies have highlighted the correlation between cervicovaginal microbiota and HPV infections as potential causative agents in cervical intraepithelial neoplasia (CIN) and CC [115].

The microbiota of the female genital tract, particularly bacterial colonization, have been extensively studied over the years. Numerous studies have explored the implications of a deficiency in vaginal *Lactobacillus* spp. and the presence of HPV on the etiology of cancer [116,117]. The Human Microbiome Project (HMP), initiated in 2008, also focused on the vaginal microbiome of females, identifying five distinct community state types (CST) labeled as CST I, II, III, IV, and V [112]. The dominant species within CSTs I, II, III, and V include *L. crispatus*, *L. gasseri*, *L. iners*, and *L. jensenii*, respectively. Conversely, CST IV is characterized by a decrease in *Lactobacillus* and a diverse variety of vaginal bacteriosis (BV), with *G. vaginalis*, *Megasphaera* spp., *Sneathia* spp., and *Prevotella* spp. being the most prevalent bacteria [118].

During the reproductive period, vaginal microbiota is affected by hormonal fluctuations. Notably, during menopause, a decline in estrogen levels results in a decrease in Lactobacillus spp., allowing for the colonization of anaerobic microorganisms. Women with HPV co-infection show *G. vaginalis*, *A. vaginae* diversity, and lower numbers of *Lactobacillus* spp. in cervical microbiomes [119]. CST III and IV are more prone to infection with HPV and the development of cervical cancer (CC) CST IV, including *Gardnerella* spp., *Prevotella* spp., *Atopobium* spp., *Anaerococcus* spp., *Peptostreptococcus* spp., *Fusobacterium* spp., and *Sneathia* spp. [118].

According to the results obtained from the study conducted by Mulato-Briones et al. [120], the isolates from the non-cancerous (NC) group comprised 28 different genera, whereas 44 genera were identified in the CC microbiota. The results showed that the NC group presented a predominance of *Lactobacillus* spp., in contrast to the CC group, which demonstrated a greater diversity of genera and species. The cluster analyses revealed seven microbiota clusters, with Cluster A characterized mainly by *Lactobacillus* spp. In the NC women, Cluster B determined *Lactobacillus* spp. dominance with the presence of *Staphylococcus* spp. It is obvious that the presence of *Candida* spp. among CC patients indicates a potential role for this yeast in the cancer progression. Cluster C was identified as containing anaerobic genera among CC patients, while *Staphylococcus* spp., *Corynebacterium* spp., and *Escherichia* spp., with *Enterococcus* spp., *Peptoniphulus* spp., and *Prevotella* spp., were prominent D–G clusters.

In the context of neoplastic processes, Pseudomonadota and Bacillota have been observed, especially in CC patients and other cancers. The most critical factors associated with cervical microbiota are dysbiosis, characterized by depletion of *Lactobacillus* spp. and the dominance of anaerobic genera, with the existence of HPV and *Candida* spp.

### 5.5. Vaginal Cancer

Primary vaginal cancer constitutes a relatively rare condition that is more common in postmenopausal women. It represents only 1% to 2% of all malignancies of the female genital tract and accounts for approximately 10% of all malignant neoplasms within the vagina. The majority of vaginal cancers, approximately 80%, are metastatic lesions that primarily originate from tumors in adjacent anatomical sites, notably the cervix or vulva, as well as from other neighboring organs [121]. Primary vaginal cancer is rare among young women and is correlated with an increase in high-risk HPV infections, along with factors such as immunosuppression and smoking, which serve as significant contributors to the etiology of vaginal cancers [18,122].

A healthy vaginal microbiome is typically characterized by the predominance *of Lactobacillus* spp., commonly *L. crispatus*, *L. gasseri*, *L. iners*, and *L. jensenii*, which play a crucial role in acidifying the vaginal environment and providing protection against invading pathogens. However, this microbiome can be influenced by various factors, including genetics, diet, ethnicity, reproductive age, pregnancy, estrogen levels, infections, sexual activity, and smoking [111,123,124]. Vaginal dysbiosis can potentially lead to chronic inflammation and increased neoplastic transformations. Specifically, alterations in the metabolism of amino acids, nucleotides, and lipids have been implicated in facilitating the onset of cancer [123,124]. Exploring the specific functions of microbial communities could reveal their underlying mechanisms.

The development of vaginal cancer has been associated with changes in the composition of the vaginal microbiota [18]. Zhou et al. [109] reported an increase in *A. vaginae*, *Gardnerella* spp., *Allobaculum* spp., *Clostridium* spp., and HPV-16, 52, and 58, along with a decrease in *L. iners* in patients with vaginal intraepithelial neoplasia. Rustetska et al. [125] suggest that dysbiosis may also play a causal role in inflammation linked to vulvar cancer. Their study identified bacterial species such as *F. nucleatum* and *Pseudomonas aeruginosa* within tumor tissues, indicating that these organisms might promote the progression of vulvar carcinoma.

The role of vaginal microbiota on neighboring organ cancers has been investigated. No association was found between the frequency of bacteria tested and malignancy. However, a difference in the prevalence of various species was observed in patients. Specifically, *Lactobacillus* spp. and *G. vaginalis* were less frequently present in patients with endometrial cancer [20].

The existing information regarding the direct relationship between microbiota and primary vaginal cancers is limited, likely due to the rarity of such cancers. However, it is important to note that disturbances in vaginal homeostasis can trigger various immune responses, leading to chronic inflammation, which is recognized as a known carcinogenic factor.

### 5.6. Prostate Cancer

Prostate cancer is the fourth most common cancer worldwide and the second most common in men [126,127]. The incidence of prostate cancer is increasing in parallel with the increase in the male population over the age of 50 [16]. Prostate inflammation is recognized as an important risk factor for the development of prostate cancer, especially considering that chronic inflammation increases the risk of developing malignancy [16,22]. Additional critical factors include age, ethnicity, family history of prostate cancer, and the presence of genetic mutations.

Prostate inflammation can result from many possible etiologies, including urinary reflux, bacterial or viral infections, diet, hormonal influences, and autoimmunity [128]. Chronic inflammation promotes cancer development and progression through mechanisms such as cellular proliferation, the increased production of growth factors, metabolic carcinogens, inflammatory cytokines, and DNA-damaging agents. These processes further disrupt the microbiota, which in turn affects overall health and physiology.

Studies examining the urethral flora of healthy adult and adolescent males have found the presence of staphylococci in 74–98% of cases, streptococci in 41–86%, and cutibacteria in 2–9% [129]. Opportunistic pathogens, such as *Enterobacterales*, originating from the gastrointestinal tract, and sexually transmitted microorganisms, such as *Neisseria gonorrhoeae*, *C. trachomatis*, and *Trichomonas vaginalis*, can directly infect the prostate [130]. Cavaretta et al. [131] found statistically significant increases (*p* < 0.05) in streptococci and staphylococci in tumors and peritumoral tissues compared to nontumoral tissues. In a case-control study conducted in Taiwan, the men with trichomoniasis had a significantly elevated risk of developing benign prostatic hyperplasia and prostate cancer compared to those without the infection [132].

Cohen et al. examined radical prostatectomy specimens of 34 patients diagnosed with cancer by needle biopsy, with prostate-specific antigen ≥ 4 ng/mL and without clinical symptoms of bacterial prostatitis. They reported that the predominant microorganism in 12 (35%) cases was *Cutibacterium acnes* (formerly *Propionibacterium acnes*). In this study, a statistically significant relationship was observed between the presence and degree of acute and chronic inflammation in radical prostatectomy specimens and the detection of *C. acnes* in culture. *C. acnes* is known to be a strong stimulant for the immune system, capable of provoking an inflammatory response. Moreover, this microorganism exhibits significant resistance to elimination by neutrophils and monocytes, thus contributing to the formation of chronic inflammation [133]. Studies have shown that *C. acnes* detected in prostate tissue samples mainly originates from the intestines and perineum skin [131]. However, the isolation of vaginal *C. acnes* from healthy women and those with vaginosis supports the possibility of sexual transmission [134].

Numerous studies have shown that microbial infections caused by dysbiosis of the gut and oral microbiome contribute to the development of intraprostatic inflammation and prostate cancer [135,136]. Pathogenic microbial species such as *Escherichia coli* can migrate from the gut microbiome and repeatedly infect the prostate, creating a microenvironment conducive to carcinogenesis [127]. A study evaluating the role of the gut microbiota in the pathogenesis of prostate cancer found significant differences in the gut microbiota when men with cancer were compared with men with benign prostates. While the relative abundance of *Bacteroides massiliensis* was observed in prostate cancer cases, the relative abundance of *Faecalibacterium prausnitzii* and *Eubacterium rectale* was predominantly detected in control subjects [137]. Additionally, a study reported that treatment of periodontal disease improved the clinical symptoms of prostatitis and reduced serum prostate-specific antigen levels in patients suffering from both periodontitis and prostatitis [138].

Oncogenic *TMPRSS2:ERG* (*ERG+*) gene fusions are seen in approximately 50% of prostate cancers. Inflammation stemming from infection plays a role in the formation of *ERG+* fusions. In addition to stimulating inflammation, bacteria can produce genotoxins such as colibactin, which is produced by some strains of *E. coli* and increases DNA damage as described in colorectal cancer pathogenesis. Shrestha et al. [11] demonstrated the role of chronic bacterial infections in driving gene alterations such as *ERG+* fusions that contribute to prostate carcinogenesis. Increased levels of intestinal metabolites, such as short-chain fatty acids, also contribute to the mechanisms promoting prostate cancer development. Short-chain fatty acids are produced by fermentation from dietary fibers and are a common product of the gut microbiome.

These metabolites are regulators of the immune system, influencing cytokine production, leukocyte influx, and apoptosis [139]. A study conducted in Japan by Matsushita et al. [140] found an increased abundance of short-chain fatty acid-producing bacteria, such as *Rikenellaceae*, *Alistipes*, and *Lachnospira*, in patients with advanced prostate cancer. In addition, higher levels of short-chain fatty acid-producing bacteria belonging to the Clostridiales order, such as *Subdoligranulum*, *Lachnobacterium*, and *Christensenellaceae*, were detected in the gastrointestinal tracts of these patients. Elevated concentrations of short-chain fatty acids increase insulin-like growth factor (IGF-1) in the prostate, which activates MAPK and PI3K signaling that promotes tumor growth in the prostate. Obese men exhibit higher fecal short-chain fatty acid levels and higher IGF-1 levels in prostate tissues compared to lean counterparts. Antibiotics have been found to neutralize high levels of *Rikenellaceae* and Clostridiales in mice subjected to a high-fat diet, resulting in reduced IGF-1 levels [139]. Liss et al. [141] found that bacteria associated with carbohydrate metabolism pathways were abundant in patients with prostate cancer, while the frequency of bacteria producing folate and vitamin B was significantly higher in men without prostate cancer.

As a result, numerous studies have elucidated the role of dysbiosis of the intestinal, urinary, oral, and even vaginal microbiota, as well as sexually transmitted infections, in the etiology of prostate cancer. However, it should be kept in mind that microorganisms such as *C. acnes*, which affects the immune system by increasing chronic inflammation, and *Rikenellaceae* and Clostridiales, which increase the production of short-chain fatty acids, may also play a role in the development of prostate cancer.

### 5.7. Testicular Cancer

Testicular cancer is classified as a relatively rare type of cancer; however, it is notably common among young men [84,142]. The testicular system harbors communities of microorganisms in varying proportions among individuals [143,144]. A recent study into testicular tissue and seminal plasma identified that the predominant microbial taxa within the male reproductive system include Bacillota, Pseudomonadota, Bacteroidota, and Actinomycetota [145]. The findings are consistent with the established profile of intestinal microbiota.

Based on literature, studies investigating the relationship between microbiota and testicular malignancies are very limited, and only one study was found that directly linked microbiota and testicular cancer. This study conducted by Mørup et al. [145] involved microbiome analysis of seminal samples collected from 48 young men (comprising 18 individuals with testicular germ cell tumors, or TGCT; five with germ cell neoplasia in situ, or GCNIS; and 25 controls) over the period from 2016 to 2019. The analysis identified a total of 2172 taxa. While the most dominant bacterial taxa were *Alteromonas mediterranea*, *Stigmatella aurantiaca*, *P. putida*, and *Gordonia* sp. KTR9, Falconid herpesvirus 1 and Simbu virus exhibited the highest relative abundance among the viral taxa. Interestingly, although *Enterobacteria* phage phiX174 sensu lato was detected as the taxon with the highest abundance among all taxa, no statistically significant differences were observed between groups.

When comparing the control group to patients diagnosed with TGCT and GCNIS, nine of the bacterial taxa (*Acaryochloris marina*, *Thermaerobacter marianensis*, *Thioalkalivibrio* sp. K90mix, *Paraburkholderia rhizoxinica*, *Mycoplasma hyopneumoniae*, *Polymorphum gilvum*, *Brucella pinnipedialis*, *Burkholderia* sp. YI23, and *Desulfurivibrio alkaliphilus*) were found to be significantly associated. Additionally, two of the viral taxa were identified with higher relative abundance in the TGCT+GCNIS group, specifically Halovirus HGTV-1 and *Vibrio* phage ICP3. Conversely, a reduction in relative abundance was observed for two bacterial taxa (*Kinetoplastybacterium crithidii* and *Cyanothece* spp.) and four viral taxa (*Streptomyces* phage VWB, *Sulfitobacter* phage phiCB2047-B, Lausannevirus, and *Methanothermobacter* phage psiM100).

Furthermore, a Mendelian modeling study conducted by Yin et al. [72] indicated that elevated levels of the genera *Peptostreptococcaceae* and *Romboutsia* were associated with an augmented risk of testicular cancer.

The connection between microbiota composition and genitourinary system cancers such as endometrial and bladder cancer has been well established [34,121]. However, the relationship between testicular cancer and microbiota is still unclear owing to TGCT’s embryonic origin, representing a unique entity with distinct biological, clinical, and therapeutic aspects. Until now, no direct causality has been confirmed between the microbiome and the risk or development of TGCT. The role of the microbiota in TGCT has yet to be investigated, particularly its potential involvement through the microbiome-gut-testis axis [146].

Cancer-testis antigens (CTA) are typically found only in healthy testes, but their aberrant expression has been observed in various cancers. An experiment demonstrated that extracts from *L. acidophilus* and *L. crispatus* reduced the transcriptional activity of CTA, such as ODF4, PIWIL2, RHOXF2, and TSGA10, in cancer cell lines [147]. Studies have indicated that *Lactobacillus* spp. may regulate CTA expression through epigenetic mechanisms [148].

Despite a non-related connection between the penis and microbiome, existing studies focus on the potential effects of circumcision and sexually transmitted illnesses [149,150]. This correlation remains mysterious due to the limited number of studies examining the relationship between penile malignancies and microbiota profiles.

The review on microbiota in urogenital cancers provides a comprehensive overview of the current research on the topic. The strengths of this review include its thorough analysis of the role of microbiota in various urogenital cancers, as well as its exploration of potential therapeutic interventions targeting the microbiome. Additionally, the review effectively synthesizes complex scientific information in a clear and accessible manner, making it a valuable resource for researchers and healthcare professionals in the field. However, one limitation of the review is the lack of discussion on the potential limitations and challenges in studying the microbiota in urogenital cancers, such as the variability of microbial composition among individuals and the influence of confounding factors. Overall, this review is a valuable contribution to the literature on microbiota and urogenital cancers.

## 6. Conclusions

This review provides insight into the potential power of urogenital microbiota in the future as diagnostic tools, prognostic indicators, and therapeutic targets for urogenital cancers. Advancing our understanding of the intricate relationship between the genitourinary microbiota and cancer development represents a rapidly emerging frontier in medical science. The microbial ecosystems of the urinary and genital tracts, though once thought to be relatively simple or even sterile, are now recognized as dynamic commu-nities that profoundly influence host health and disease. Dysbiosis in these microbiomes has been repeatedly implicated in the initiation, progression, and treatment outcomes of various cancers, including bladder, kidney, cervical, ovarian, and endometrial cancers.

In healthy individuals, the dominance of protective microbial species such as *Lactobacillus* in the vaginal and urinary microbiota contributes to maintaining a low pH, preventing pathogen colonization, and regulating immune responses. However, cancer-related microbial imbalances often exhibit reduced levels of *Lactobacillus* and increased microbial diversity, leading to inflammation, genotoxicity, and metabolic disruptions. In bladder cancer, for instance, studies have linked microbial dysbiosis to chronic inflammation and carcinogenesis, particularly in regions endemic to infections such as Schistosoma haematobium. Similarly, kidney cancer has been associated with distinct microbial profiles in both urinary and gastrointestinal systems, reflecting the broader systemic impact of microbiota alterations.

The gut microbiota also emerges as a critical player, influencing genitourinary cancers through systemic effects on hormone regulation, immune modulation, and the production of metabolites like short-chain fatty acids. These interactions underscore the interconnectedness of the body’s microbial ecosystems, where changes in one site, such as the gut, can ripple through to affect distant organs like the bladder, kidneys, and reproductive tract.

Of particular significance is the concept of the “oncobiome,” which emphasizes the role of microbial genetic material in promoting or suppressing cancer. Advanced micro-biome analyses have identified specific microbial signatures that correlate with different cancer types, providing a foundation for novel diagnostic and therapeutic approaches. For example, certain microbial taxa, such as *A. muciniphila* and *Bacteroides* spp., have been associated with improved treatment responses in renal cancer, highlighting the potential of microbiota as predictors of therapeutic outcomes.

This growing body of evidence underscores the urgent need for multidisciplinary research that integrates microbiology, oncology, genomics, and immunology. Future studies should aim to unravel the causal pathways between microbiota dysbiosis and cancer, explore the role of microbial metabolites and immune interactions, and develop microbiota-based therapies. Innovations such as probiotics, fecal microbiota transplants, and microbiome-informed drug development may transform the landscape of cancer prevention and treatment.

Ultimately, a deeper understanding of the genitourinary microbiome holds the promise of shifting cancer management from reactive to preventive, empowering clinicians with tools to diagnose cancers earlier, tailor treatments more effectively, and improve the quality of life for millions of patients worldwide. The microbiota, once a neglected aspect of human biology, is poised to become a cornerstone of personalized oncology.
